# Supplementary material for: Coupling of Polo kinase activation to nuclear localization by a bifunctional NLS is required during mitotic entry
Source: Nat Commun. 2017 Nov 22;8:1701. doi: 10.1038/s41467-017-01876-8 (PMC5700101; doi:10.1038/s41467-017-01876-8)

Supplementary Fig. 1

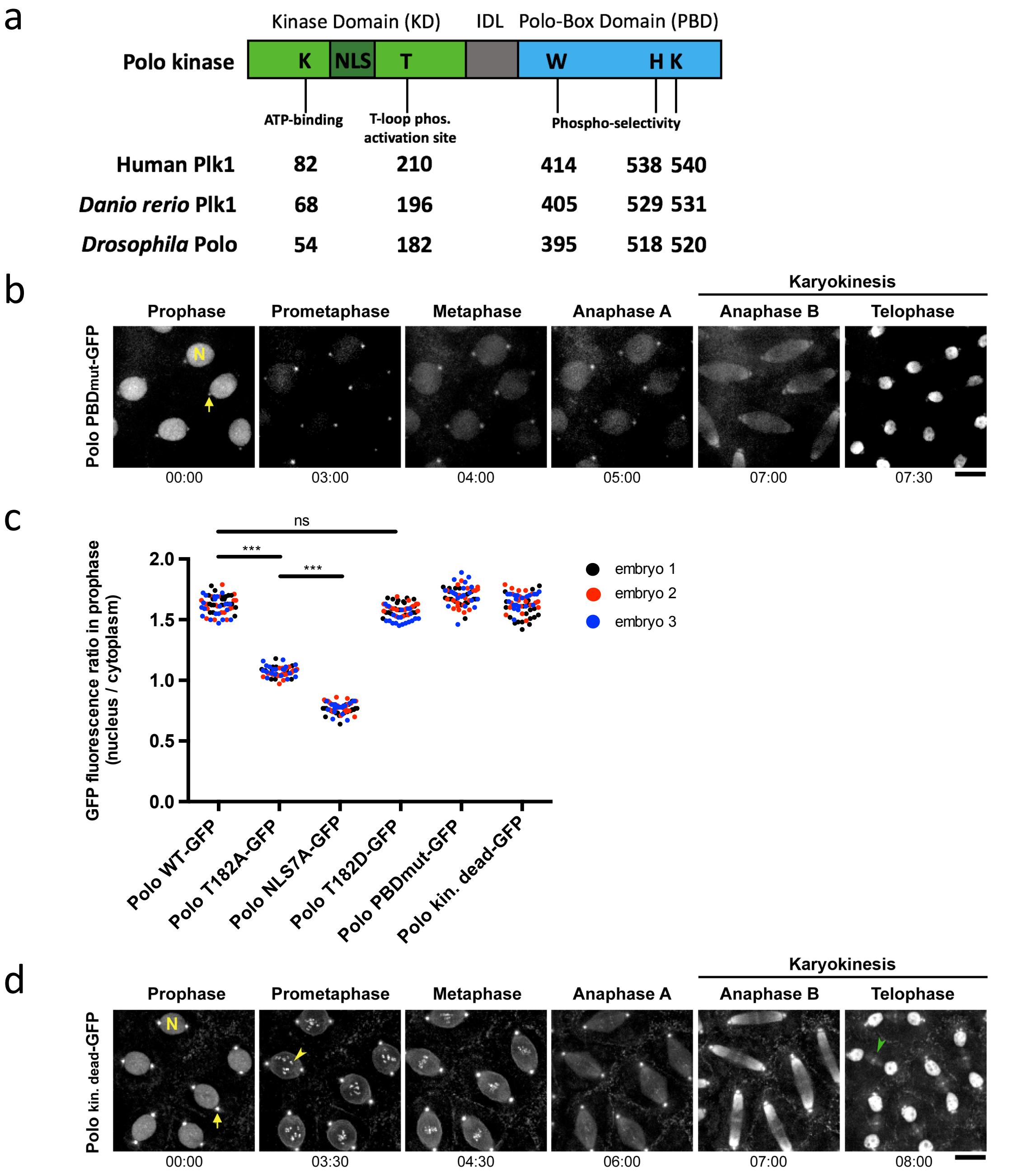

Supplementary Fig. 1 Expression of different forms of Polo-GFP in the early embryo. a. Schematic representation of the Polo kinase in three species. The positions of the Kinase Domain (KD, green), the Polo-Box Domain (PBD, cyan), the Inter-Domain Linker (IDL, grey) and the Nuclear Localization Signal (NLS, dark green) are depicted. Residues that are crucial for ATP-binding, enzymatic activation (activation loop) and phospho-selectivity are indicated. **b.** Syncytial embryos expressing Polo^{PBDmut}-GFP were observed by time-lapse microscopy. N: a nucleus; arrow: a centrosome. Bar: 10 µm. **c.** Quantification of the nucleus/cytoplasm ratios of GFP fluorescence intensity in syncytial embryos expressing different forms of Polo-GFP. Twenty nuclei were quantified in 3 different embryos in each case. **d.** Syncytial embryos expressing Polo$^{kin.\ dead}$-GFP were observed by time-lapse microscopy, as in (b). Yellow arrowhead: centromere/kinetochore; green arrowhead: midbody ring. Bar: 10 µm.

Supplementary Fig. 2

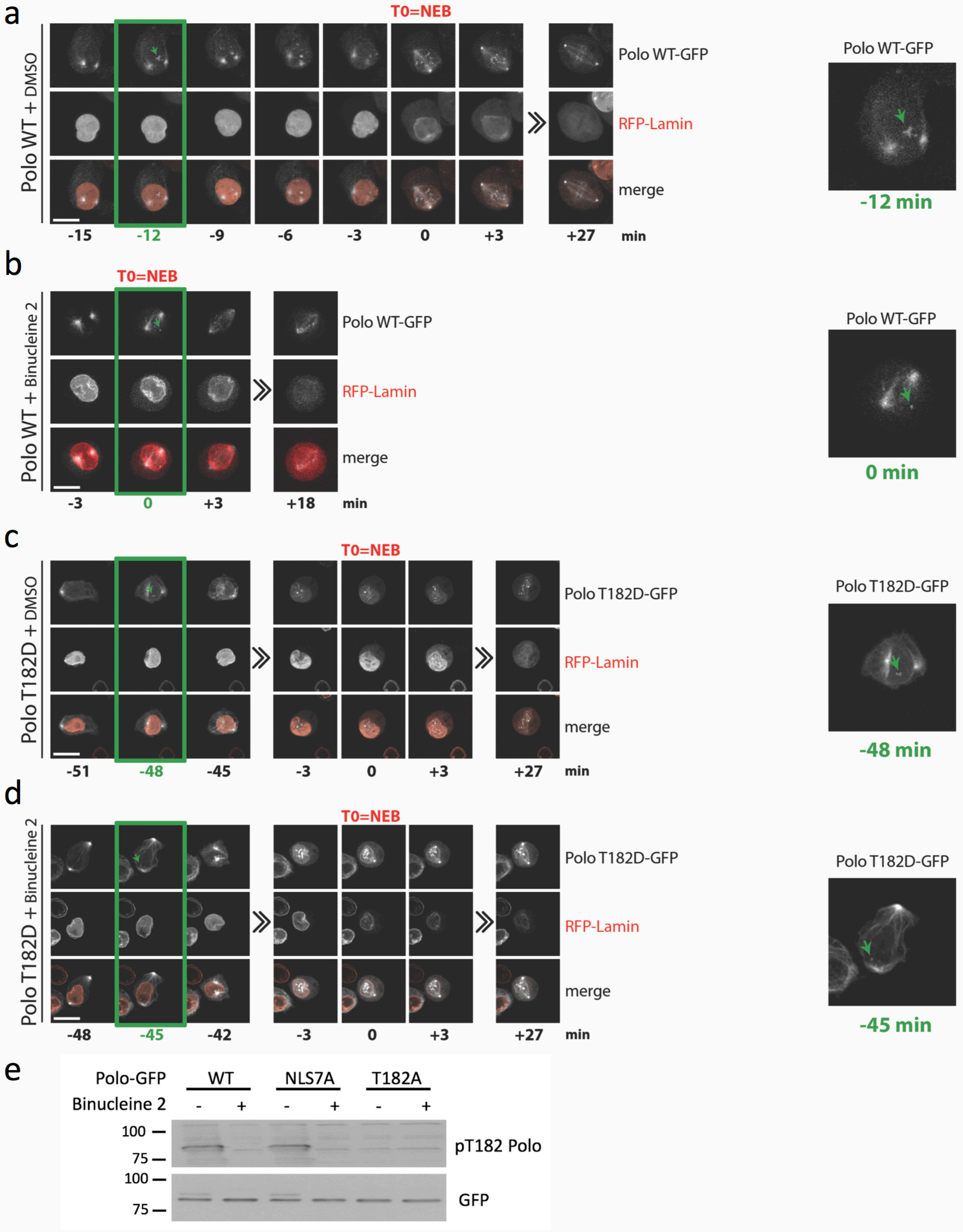

Supplementary Fig. 2 The nuclear accumulation of Polo during prophase depends on its phosphorylation at the activating site. a-d. Cells expressing $Polo^{WT}$-GFP or $Polo^{T182D}$-GFP were treated with Binucleine 2 (Aurora B inhibitor) or with DMSO (control) and were filmed. See Fig. 1d for quantification of fluorescence. **e.** Inhibition of Aurora B with Binucleine 2 abrogates Polo phosphorylation at Thr182. Cells were treated with 100 nM of okadaic acid to inhibit phosphatases for 1 hr before lysis. Westerns blots were probed as indicated.

Supplementary Fig. 3

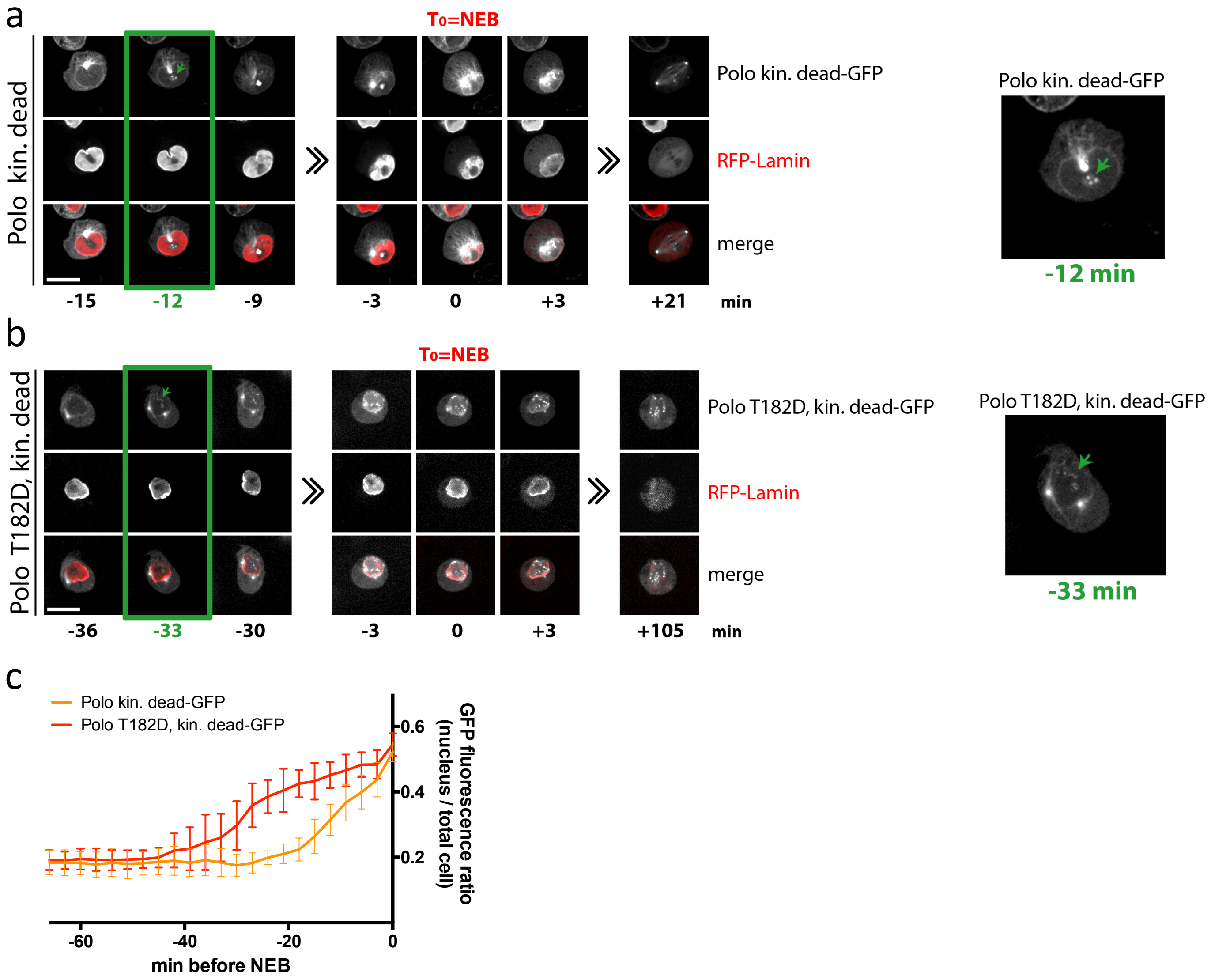

Supplementary Fig. 3 The advancement of Polo nuclear localization caused by the T182D substitution does not depend on Polo activity. a, b. Time-lapse imaging of cells expressing Polo$^{\text{kin. dead}}$-GFP or Polo$^{\text{T182D, kin. dead}}$-GFP with RFP-Lamin. Bar: 5 µm. We noted that Polo$^{\text{T182D, kin. dead}}$-GFP is highly toxic, causing mitotic failures, possibly because of a PBD-dependent dominant negative effect. **c.** The GFP fluorescence ratio (nucleus/total cell) was measured in the period preceding NEB (n=10, error bars: SD).

Supplementary Fig. 4

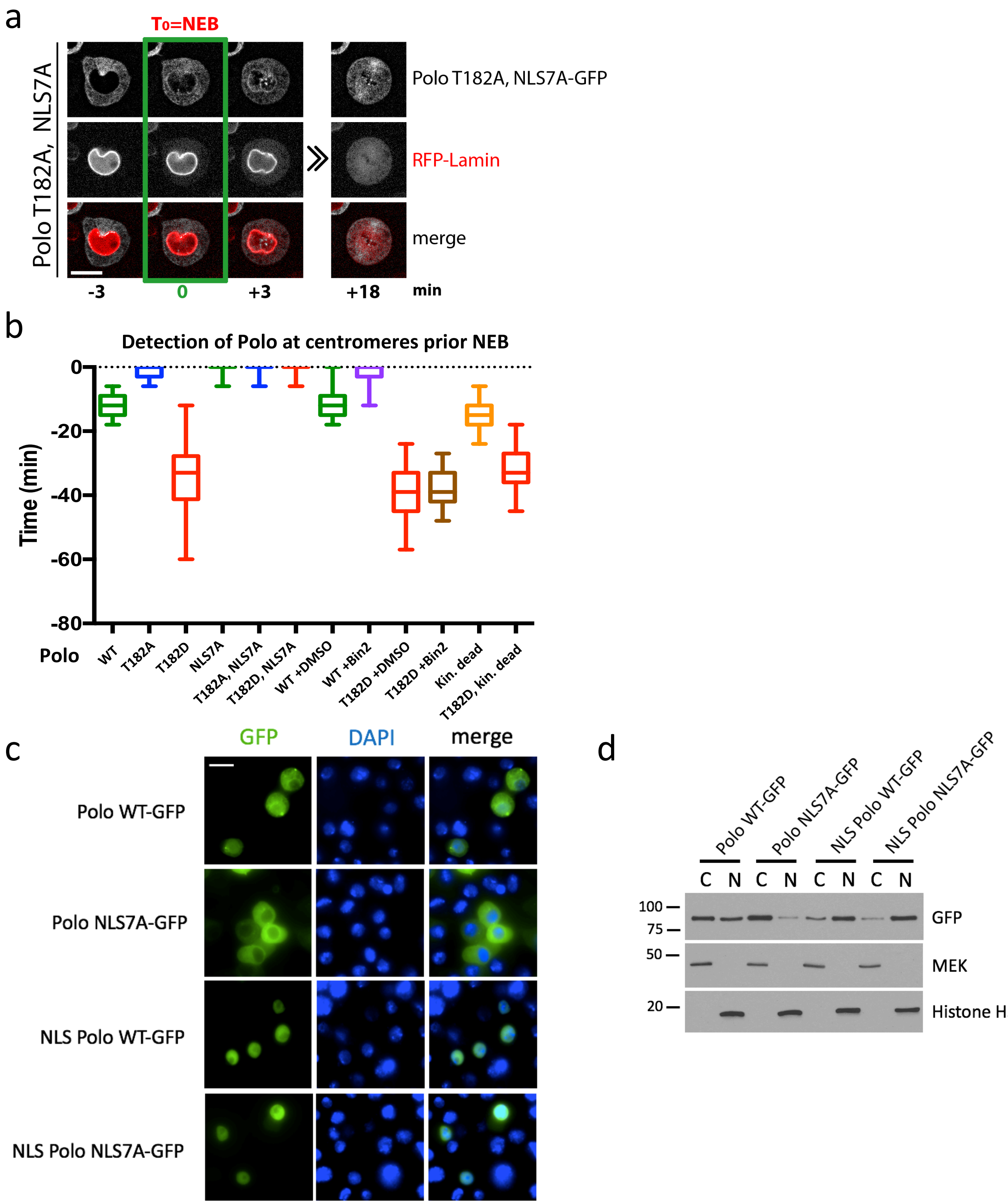

Supplementary Fig. 4 Nuclear accumulation of Polo depends on an NLS. a. Time-lapse imaging of a cell expressing Polo$^{T182A,\ NLS7A}$-GFP with RFP-Lamin. Bar: 5 µm. **b.** Box plot indicating the time between the initial detection of Polo^{WT}-GFP at centromeres and NEB (T_0) (n=40, repeated three times). The upper and lower edge of each box represents upper and lower quartiles, respectively. The median is indicated by a horizontal line and lines extending from each end of the box indicate the peak values. **c, d.** N-terminal fusion of the Polo NLS motif (LCKKRSMMELHKRRK) makes Polo-GFP constitutively nuclear. The localization was examined by microscopy on fixed cells (c) and by cell fractionation followed by Western blots (d).

Supplementary Fig. 5

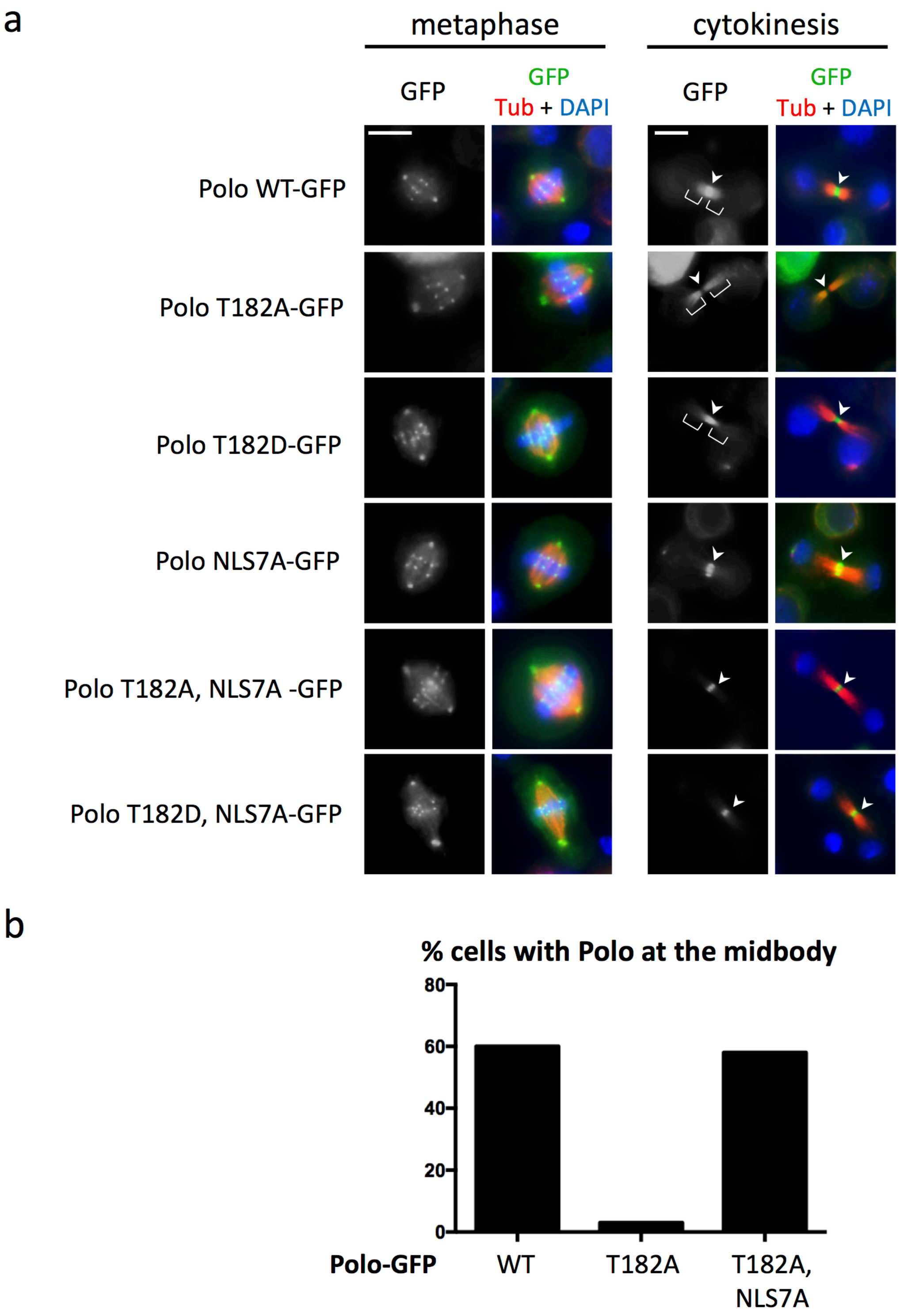

Supplementary Fig. 5 Localization of Polo-GFP WT and mutants during metaphase and cytokinesis. a. Immunofluorescence. Brackets: central spindle microtubule localization of Polo-GFP, dependent on its interaction with Map205. Arrowhead: midbody ring localization of Polo-GFP requires Polo to be free from Map205. Bar: 5 µm. **b.** Percentage of cells with Polo at the midbody during cytokinesis are indicated (n=100). Mutation of the NLS largely abrogates the localization of Polo-GFP to microtubules and favors its localization to the midbody ring, consistent with the abrogation of its interaction with Map205 (Fig. 3b).

Supplementary Fig. 6

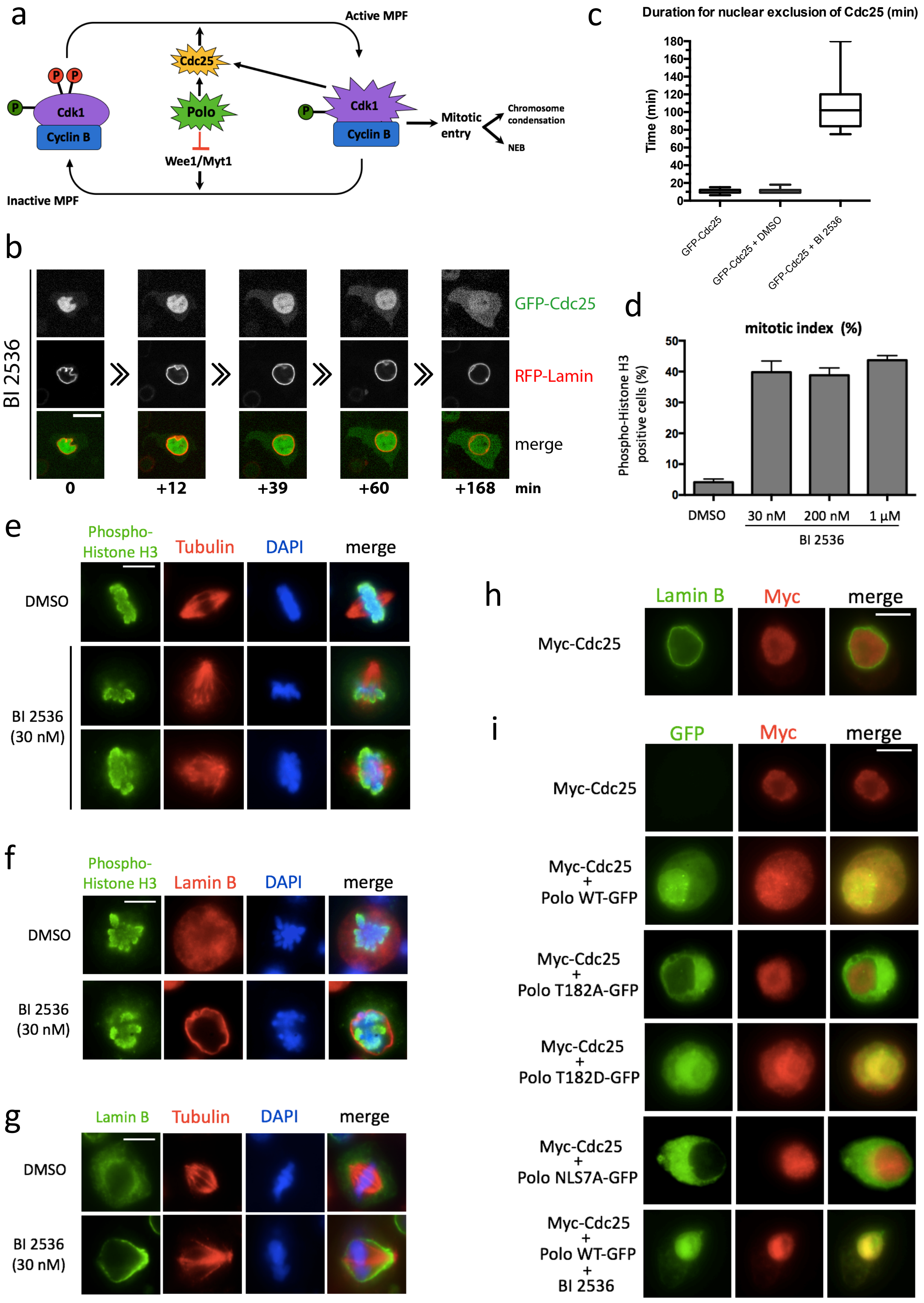

Supplementary Fig. 6 Polo regulates Cdc25. a. Model for Polo and Cdc25-dependent activation of Cyclin B-Cdk1 at mitotic entry. During interphase, Cdk1 is maintained in an inactive state through inhibitory phosphorylations at Thr14 and Tyr15 (red) by the Wee1 and Myt1 kinases. At mitotic entry, dephosphorylation of these residues by the Cdc25 phosphatases activates Cyclin B-Cdk1 which induces chromosome condensation and NEB. To promote this process, Polo phosphorylates and activates Cdc25, and phosphorylates and inactivates Wee1 and Myt1. **b, c.** Inhibition of Polo kinase (30 nM BI 2536 added at T_0) extends the time during which the cytoplasmic localization of GFP-Cdc25 increases. In the presence of BI 2536, GFP-Cdc25 never becomes more concentrated in the cytoplasm than in the nucleus (compare with Fig. 4b). At least 30 cells were scored for each condition in two independent experiments. We could not quantify the timing of changes in fluorescence relative to NEB because NEB did not occur in these cells. **d.** Mitotic index of cells treated with different concentrations of BI 2536 during 16 hours. Results from three independent experiments were combined (n=300). Error bars: SD. **e-g**. Inhibition of Polo kinase leads to spindle defects and hampers Lamin disassembly in mitotic cells. Immunofluorescence in cells treated with BI 2536 (30 nM) or DMSO. Bar: 5 µm. **h.** Myc-Cdc25 is a nuclear protein. Immunofluorescence for Lamin B and Myc. Bar: 5 µm. **i**. Polo activity in the nucleus promotes the cytoplasmic localization of Cdc25. Immunofluorescence in cells overexpressing Myc-Cdc25 and different forms of Polo-GFP or treated with BI 2536 (30 nM). Bar: 5 µm.

Supplementary Fig. 7

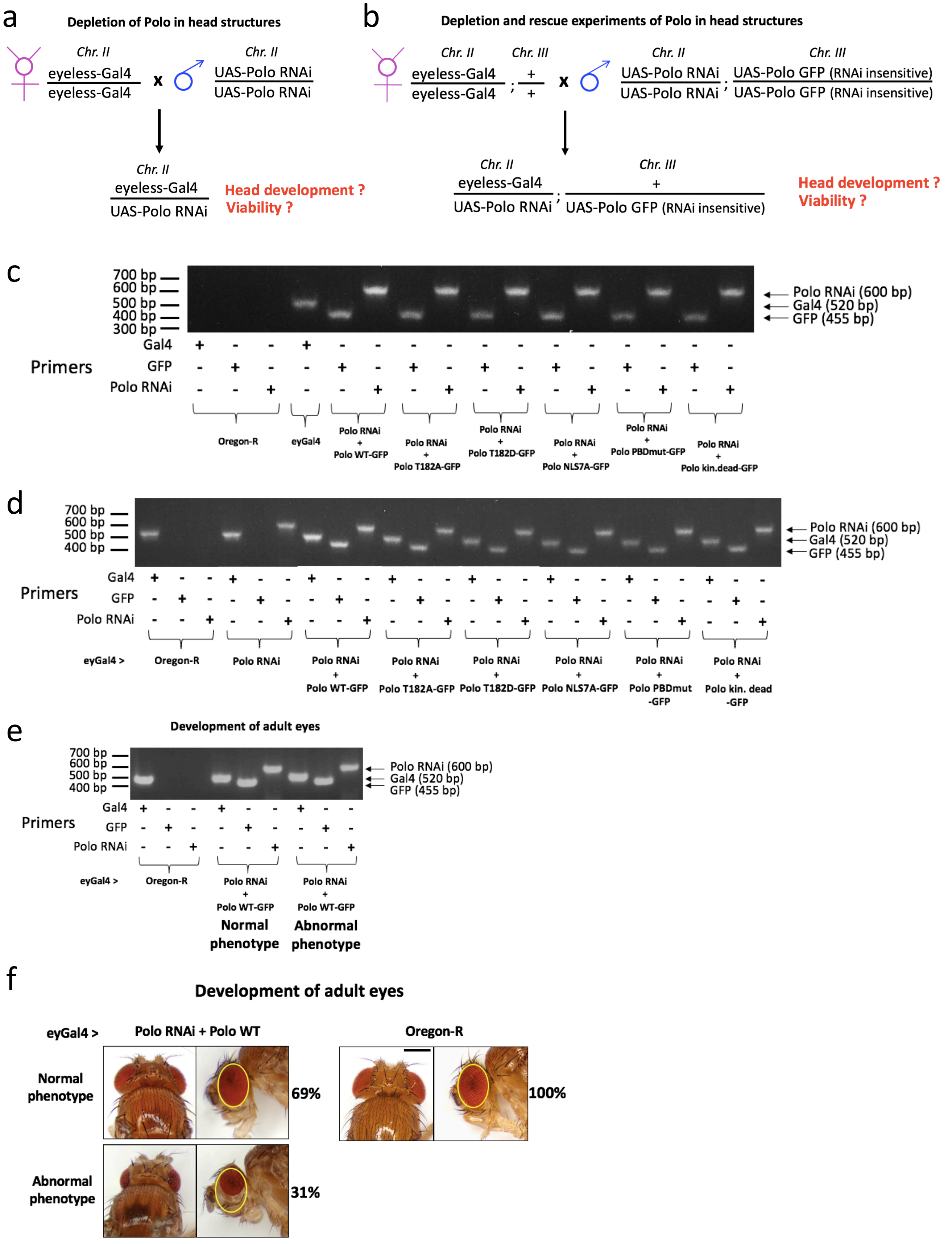

Supplementary Fig. 7 Genetic manipulations and validations for rescue experiments in the head. a. Crossing scheme for depletion of Polo in the developing head. **b.** Crossing scheme for simultaneous depletion of endogenous Polo and expression of RNAi insensitive forms of Polo in the developing head. **c.** PCR amplification of DNA isolated from flies (parents) used for the cross described in (b). To test the presence of our genetic constructs, we PCR-amplified sequences specific to each transgene. The length (in bp) of each sequence amplified for each transgene is indicated. **d, e.** PCR amplification of DNA isolated from progeny flies resulting from the cross described in (b). **f.** In flies where $Polo^{WT}$-GFP rescues from loss of endogenous Polo, heads and eyes are sometimes abnormally small. Quantifications are indicated (n=300).

Supplementary Fig. 8

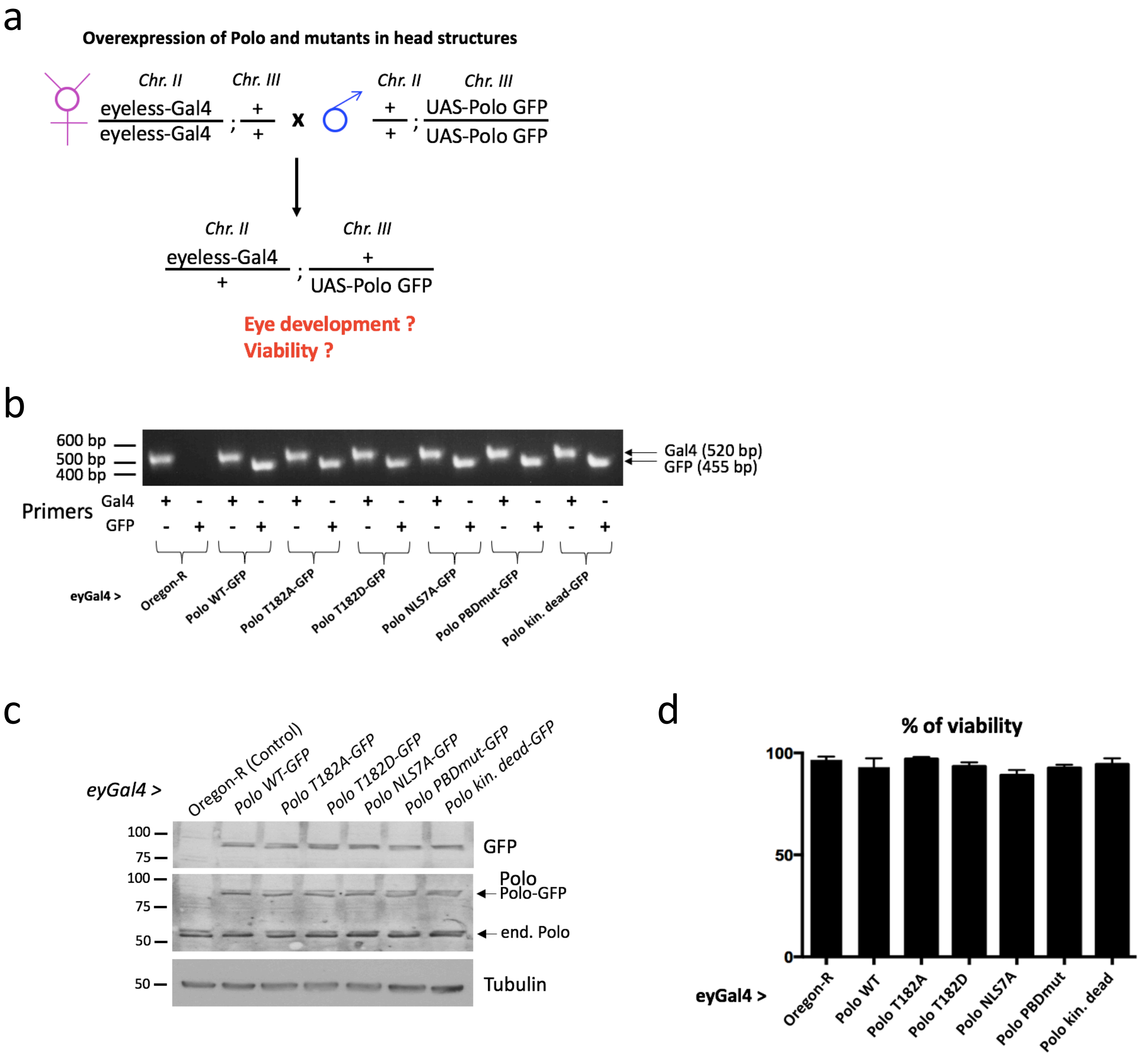

Supplementary Fig. 8 Genetic manipulations and validations for overexpression of different forms of Polo-GFP in the head. a. Crossing scheme for overexpression of Polo and mutants in the developing head. **b.** PCR amplification of DNA isolated from flies resulting from the cross described in (a). **c.** Different forms of Polo-GFP are expressed at similar levels from transgenes driven by *ey-Gal4* in larvae. Western blots are shown. **d.** Quantifications of viability for each genotype (n=300). Error bars: SD.

Supplementary Fig. 9

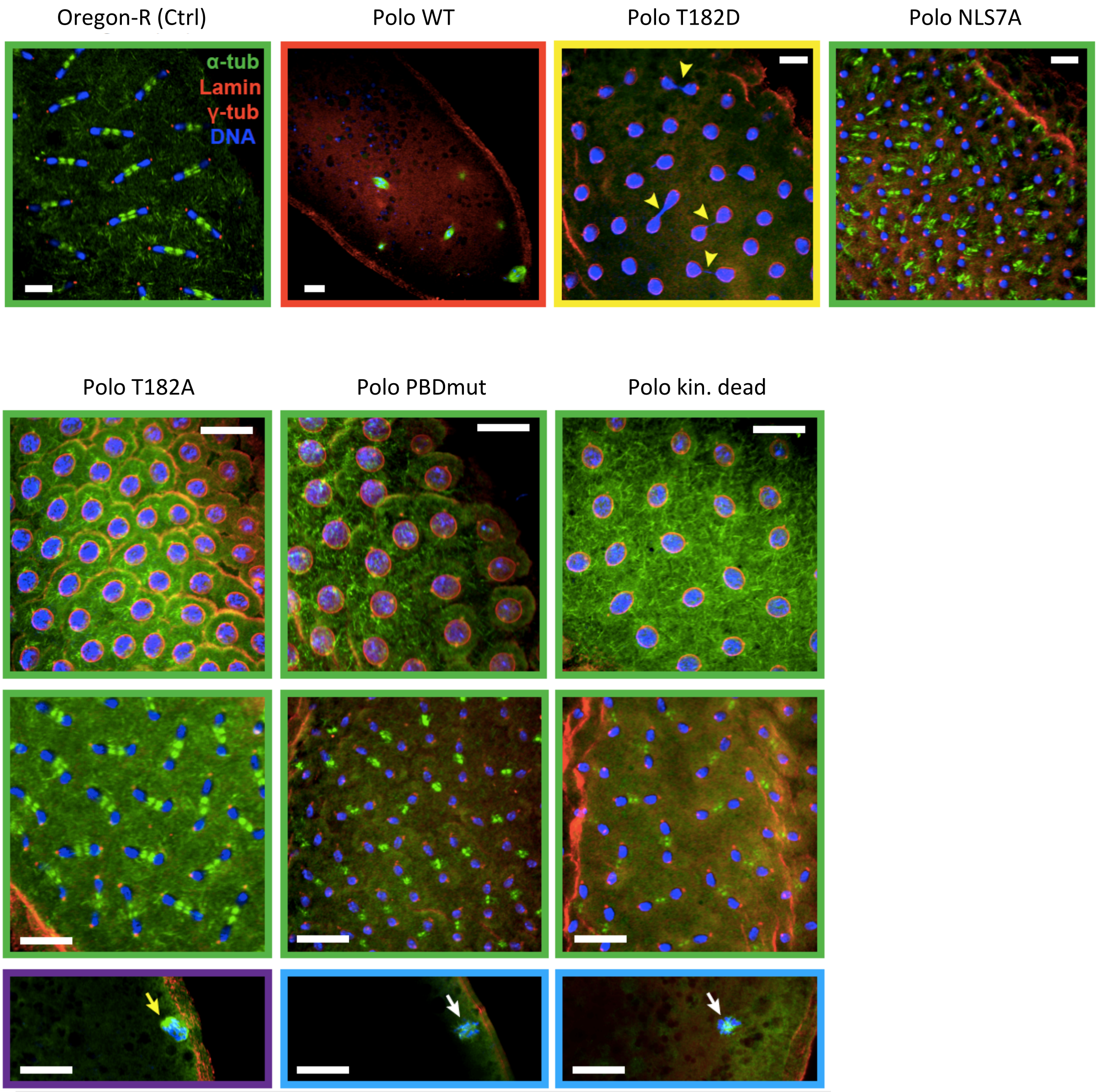

Supplementary Fig. 9 Phenotypes resulting from expression of different forms of Polo-GFP in eggs and embryos (additional images in complement to Fig. 6f, g). Eggs and embryos laid by females expressing different forms of Polo-GFP were collected every 2 hrs and phenotypes were examined by immunofluorescence. After fixation, the GFP was not visible. The different categories are color-coded as in Fig. 6f, g. Yellow arrowheads: anaphase defects. White arrows: polar body indicated normally completed meiosis. Yellow arrows: meiosis spindles blocked in metaphase I. Scale bars: 20 mm.

Uncropped western blots from primary figures

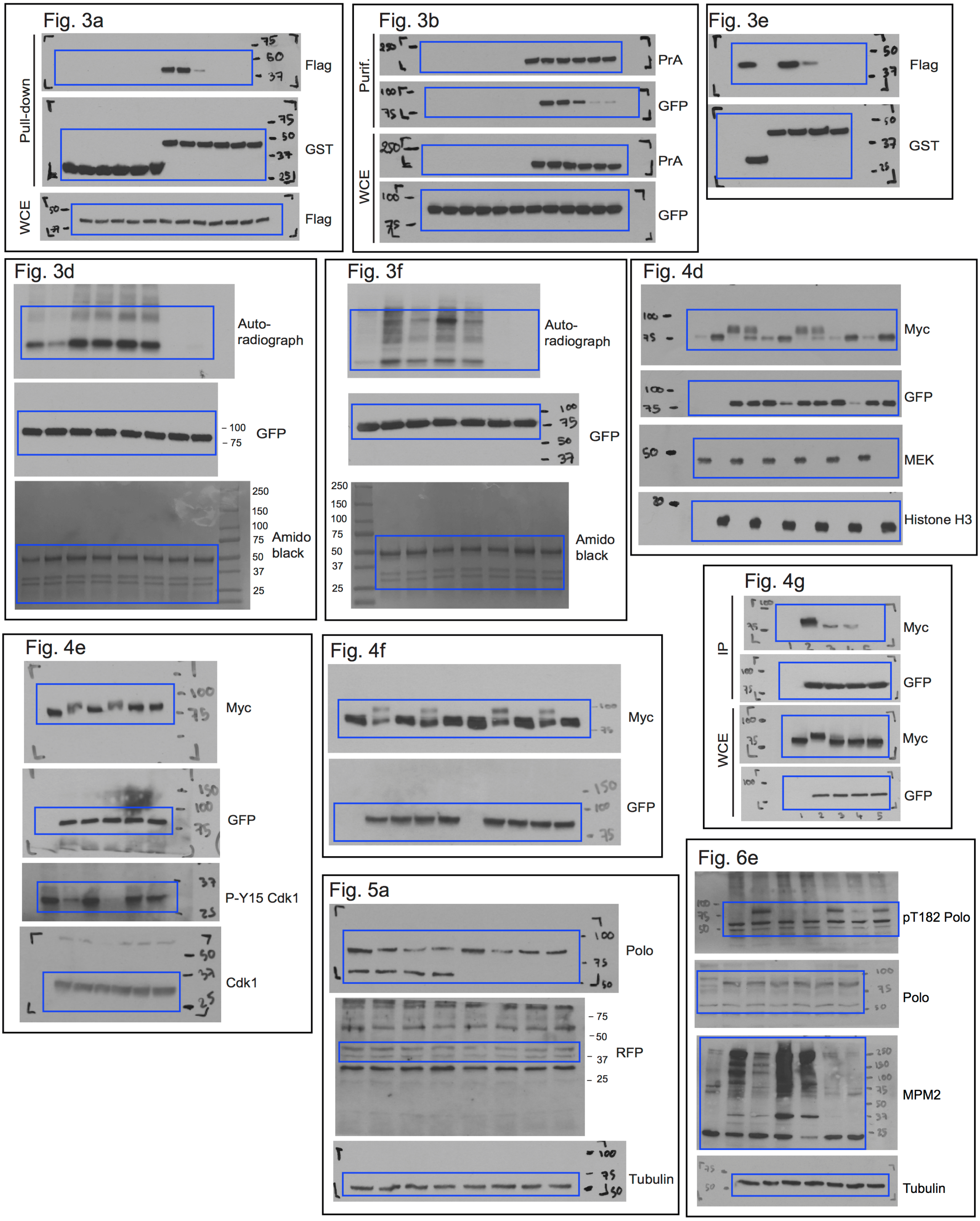

Uncropped western blots from supplementary figures

Supplementary Fig. 2e

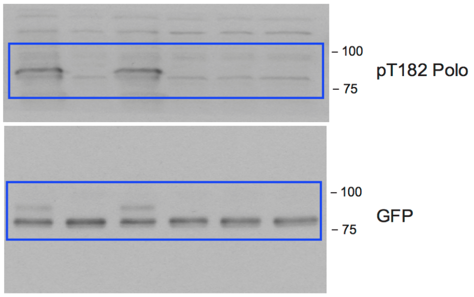

Supplementary Fig. 4d

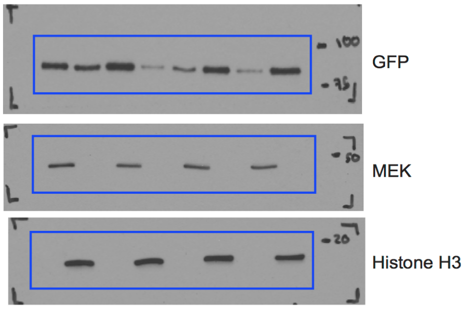

Supplementary Fig. 8c

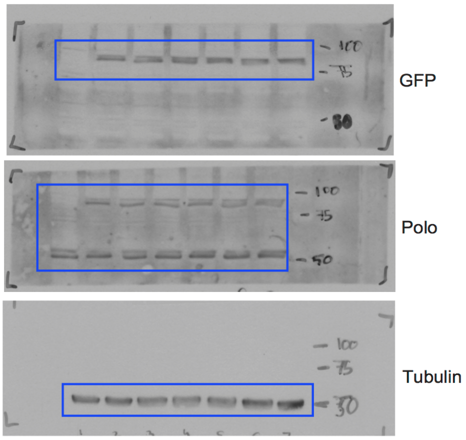

Supplement: Supplementary file 1 — Supplementary Information [file 41467_2017_1876_MOESM1_ESM.pdf]
