# Supplementary material for: Coupling of Polo kinase activation to nuclear localization by a bifunctional NLS is required during mitotic entry
Source: Nat Commun. 2017 Nov 22;8:1701. doi: 10.1038/s41467-017-01876-8 (PMC5700101; doi:10.1038/s41467-017-01876-8)
Supplement: Supplementary file 2 — Descriptions of Additional Supplementary Files [file 41467_2017_1876_MOESM2_ESM.pdf]

Descriptions of Additional Supplementary Files

File Name: Supplementary Movie 1
Descriptions: Time-lapse imaging of a Drosophila syncytial embryo expressing PoloWT-GFP. Pictures were acquired every 30 sec on a spinning disk confocal microscope. Bar: 10 µm.

File Name: Supplementary Movie 2
Descriptions: Time-lapse imaging of a Drosophila syncytial embryo expressing PoloT182A-GFP. Pictures were acquired every 30 sec on a spinning disk confocal microscope. Bar: 10 µm.

File Name: Supplementary Movie 3
Descriptions: Time-lapse imaging of a D-Mel cell expressing PoloWT-GFP and RFP-Lamin. Pictures were acquired every 3 min on a spinning disk confocal microscope. T0=NEB. Bar: 5 µm.

File Name: Supplementary Movie 4
Descriptions: Time-lapse imaging of a D-Mel cell expressing PoloT182A-GFP and RFP-Lamin. Pictures were acquired every 3 min on a spinning disk confocal microscope. T0=NEB. Bar: 5 µm.

File Name: Supplementary Movie 5
Descriptions: Time-lapse imaging of a D-Mel cell expressing PoloT182D-GFP and RFP-Lamin. Pictures were acquired every 3 min on a spinning disk confocal microscope. T0=NEB. Bar: 5µm.

File Name: Supplementary Movie 6
Descriptions: Time-lapse imaging of a Drosophila syncytial embryo expressing PoloNLS7A-GFP. Pictures were acquired every 30 sec on a spinning disk confocal microscope. Bar: 10 µm.

File Name: Supplementary Movie 7
Descriptions: Time-lapse imaging of a D-Mel cell expressing GFP-Cdc25 and RFP-Lamin. Pictures were acquired every 3 min on a spinning disk confocal microscope. T0=NEB. Bar: 5µm.

File Name: Supplementary Movie 8
Descriptions: Time-lapse imaging of a D-Mel cell expressing GFP-Cdc25 and PoloWT-RFP. Pictures were acquired every 3 min on a spinning disk confocal microscope. T0=NEB. Bar: 5 µm.
